# Patient-Reported Outcome Measures in Cystic Fibrosis: Protocol for a Systematic Review

**DOI:** 10.2196/15467

**Published:** 2020-05-06

**Authors:** Irushi Ratnayake, Susannah Ahern, Rasa Ruseckaite

**Affiliations:** 1 Department of Epidemiology and Preventive Medicine Monash University Melbourne Australia

**Keywords:** patient-reported outcome measure, PROM, cystic fibrosis, health-related quality of life

## Abstract

**Background:**

Patients with cystic fibrosis (CF) can struggle with burdensome symptoms and treatment regimens that negatively affect every aspect of their life. As physiological parameters can fail to capture these complications, the assessment of health-related quality of life (HRQOL) has gained prominence. HRQOL can be measured using standardized patient questionnaires called patient-reported outcome measures (PROMs). The Australian Cystic Fibrosis Data Registry (ACFDR) collects clinical data on adult and pediatric patients with CF. The incorporation of PROMs into the ACFDR would enable monitoring of HRQOL trends, benchmarking of HRQOL outcomes, and support of HRQOL research in CF.

**Objective:**

Prior to incorporation of a PROM in the ACFDR, this systematic review was planned to evaluate whether any suitable PROMs are currently being used for CF.

**Methods:**

This systematic review will be conducted in compliance with the PRISMA-P (Preferred Reporting Items for Systematic Reviews and Meta-Analyses Protocols) guidelines. MEDLINE, EMBASE, Scopus, CINAHL (Cumulative Index of Nursing and Allied Health Literature), PsycINFO, and Cochrane Library databases were searched for articles published between January 2009 and February 2019 on the use of PROMs to measure HRQOL in adult and pediatric patients with CF. Study designs such as observational studies, reviews and validation studies were included. Studies describing randomized controlled trials, dissertations, books, guideline statements, and abstracts were excluded. The COnsensus-based Standards for the selection of health Measurement INstruments (COSMIN) risk of bias checklist was used to assess the methodological quality of included studies. A descriptive synthesis of the results will be undertaken in line with the outcomes of this study.

**Results:**

As of July 2019, the search has been conducted and 4530 records were screened. After two phases of screening, 97 studies were included in the final review and subjected to data extraction. Reviewers are currently in the process of critical appraisal.

**Conclusions:**

This review will identify any PROM(s) that may be used to measure HRQOL in the ACFDR.

**Trial Registration:**

PROSPERO International Prospective Register of Systematic Reviews CRD42019126931; https://www.crd.york.ac.uk/prospero/display_record.php?RecordID=126931

## Introduction

### Disease Background

Cystic fibrosis (CF) is the most common life-shortening autosomal recessive disease affecting Caucasian populations [1]. CF (ICD-10 code E84) is caused by mutations affecting the cystic fibrosis transmembrane conductance regulator (CFTR) protein, which transports chloride ions across epithelial cell membranes [2]. Changes in chloride ion concentration cause thicker exocrine secretions and increased salt concentration throughout the body [1]. In the respiratory tract, where the disease is most detrimental, thickened mucus restricts the airway lumen [2] and impairs clearance of microorganisms, resulting in chronic cough, increased infections, and bronchiectasis [3]. Pulmonary disease can progress to respiratory failure and death [2]. Other common consequences of CFTR mutations include pancreatic insufficiency, impaired intestinal motility, impaired growth, and diabetes [2].

Although life expectancy with CF has improved significantly in the last few decades [4], patients with CF continue to struggle with symptoms that have a profound impact on all areas of life [5]. In addition, daily treatment regimens have become more complex and time-consuming and can require 2-3 hours a day [6]. Traditional physiological parameters that measure disease severity do not capture the impact of symptom and treatment burden on daily functioning. As a result, the assessment of health-related quality of life (HRQOL) in CF has gained prominence as an alternative measure of disease severity and functional limitation [7].

HRQOL has been defined as “an individual’s perception of their position in life” [8]. It is a multidimensional construct that encompasses physical symptoms, daily functioning, psychological well-being, social functioning, and relationships [8]. As these domains are best understood and described by patients themselves, patient-reported outcome measures (PROMs) are commonly used to report HRQOL [6]. PROMs are standardized questionnaires filled out by patients or their proxies [9]. They capture patients’ perceptions of their own well-being [9].

In CF, PROMs are currently used for a variety of purposes including the following: as outcomes in clinical trials, to evaluate the efficacy of new interventions, to measure the effects of disease on patient functioning, or to compare the cost-effectiveness of treatments [10]. However, PROMs can be used most effectively when captured prospectively and longitudinally through routine data collection [11]. Including a PROM within a pre-existing clinical registry is a cost-effective method of HRQOL data collection [11]. When PROMs have been incorporated in national [12] and international [11,13,14] registries for other diseases, they have been used to track treatment outcomes, monitor HRQOL trends, and support benchmarking and quality improvement [15].

### Australian Cystic Fibrosis Data Registry

The Australian Cystic Fibrosis Data Registry (ACFDR) was established in 1998 and collects clinical data on adult and pediatric patients with CF. Information is collected multiple times a year from specialist clinics [16]. At the end of 2017, the ACFDR held data from 3151 patients [16], estimated to be over 90% of Australian patients with CF [17]. Data collected included patients’ demographics, social functioning, physical health, treatments, hospitalizations, and mortality [16]. Growing interest in the incorporation of PROMs in Australian registries [18] has led to the evaluation of a HRQOL PROM in the ACFDR. This systematic review was planned as the first phase of a project to identify a PROM that would be appropriate to include in ACFDR data collection.

### Objectives

Preliminary searches identified no published data on the use of PROMs in CF clinical registries and no recent systematic reviews summarizing adult and pediatric HRQOL PROMs in CF. Therefore, we planned a systematic review to examine all PROMs currently applied to CF populations to identify whether any PROMs are suitable to incorporate in the ACFDR. We require information on the populations and contexts PROMs are used, their reliability and validity in those populations, and how they are perceived by patients. Information on mode and frequency of PROMs administration is also required. We believe this systematic review will identify a suitable PROM to use in the ACFDR and the best method to implement this PROM.

The primary objective of the proposed systematic review is to identify which PROMs examining HRQOL have been used in adult and pediatric populations with CF and to summarize their psychometric properties. Secondary objectives are to identify how PROMs are administered and assess patient perceptions of PROMs.

## Methods

This systematic review protocol follows the PRISMA-P (Preferred Reporting Items for Systematic Review and Meta-Analyses Protocol) guidelines [19]. A detailed description on population, intervention, comparison, and outcome of the systematic review is outlined in Textbox 1.

### Inclusion and Exclusion Criteria

Inclusion and exclusion criteria for articles can be found in Textbox 2.

**Population, intervention, comparison, and outcome (PICO) of systematic review.**

**Population:** Adults (aged 18 years old and above) and children (aged under 18 years old) with diagnosed cystic fibrosis (CF)

**Intervention:** Generic and disease-specific patient-reported outcome measures (PROMs) that evaluate health-related quality of life in patients with CF

**Comparison:** Studies without a comparator will be considered for inclusion.

**Outcome:** The primary outcome measure is the assessed or stated psychometric properties of PROMs. The secondary outcome measures are (1) contexts in which PROMs have previously been used, (2) administration methods of PROMs, and (3) acceptability of PROMs for patient population.

**Article inclusion and exclusion criteria.**

**Inclusion Criteria:**

- Articles describing the use of patient-reported outcome measures (PROMs) to measure health-related quality of life in cystic fibrosis (CF)
- Study participants of all ages and genders with a prior diagnosis of CF, including cases where proxy respondents have completed PROMs on behalf of patients
- Study designs including reviews, observational studies, and validation studies
- Available in English language
- Published in the last decade (from January 2009 to February 2019)

**Exclusion Criteria:**

- Published before January 2009
- Describing randomized control trials
- Unpublished manuscripts, dissertations, books and book chapters, conference proceedings, meeting abstracts, and guideline statements

### Study Design

Quantitative (eg, cohort, longitudinal, prospective, retrospective, validation, and case studies) and qualitative studies (eg, phenomenological, grounded theory, and case reports) exploring HRQOL outcomes in patients with CF were included. Mixed methods research articles were also included in the review.

### Context

Studies conducted in clinical environments such as acute care (hospital inpatient services and emergency departments) and subacute care (primary health care and outpatient clinics) were included. Patients recruited from databases, patient support groups, and registries were also included.

### Outcomes of Interest

The primary outcome of interest is to identify which PROMs are currently used in adult and pediatric populations with CF and to summarize the psychometric properties of PROMs (eg, content validity, internal consistency, responsiveness), as assessed for the study population or as stated based on previous studies.

Secondary outcome measures include (1) contexts in which HRQOL PROMs are currently used (eg, interventional studies, prevalence studies, clinical registries); (2) administration methods of PROMs (eg, paper survey, electronic, interview, use of proxy-respondents); and (3) acceptability of PROMs (eg, relevance, ease of use, clarity) as described by authors of the study.

### Search Methods

Initial Ovid MEDLINE searches were undertaken to find published studies and reviews relevant to the topic. Keywords and index terms from these articles were recorded and used to develop the final search strategy. The search strategy was finalized in Ovid MEDLINE and adapted as required for other databases using the MeSH trees. The draft search strategy included the terms “patient reported outcome” OR “patient reported outcome measure” OR “self-report*” OR “questionnaire” OR “scale” OR “perception” OR “quality of life” OR “QOL” AND “cystic fibrosis.” The search was restricted to the past 10 years to only include PROMs relevant to the current population with CF.

The following databases were searched: MEDLINE, EMBASE, Scopus, CINAHL (Cumulative Index of Nursing and Allied Health Literature), PsycINFO, and Cochrane Library. A search of the gray literature was not conducted. Bibliographies of all selected studies fulfilling inclusion criteria will be scanned to identify any articles missed by the search.

### Data Management

All studies identified in database searches were compiled in Endnote X7 (Clarivate Analytics). Duplicates were deleted using the Endnote “Remove Duplicates” function and a manual scan of the results. Review documentation and search results were saved and backed up in Monash University’s faculty-allocated network storage (S-drive). Data will only be accessed by the reviewers.

### Selection Process

In the first stage of screening, one reviewer (IR) read the titles and abstracts of all studies identified by the search. Studies that met the inclusion criteria were included. During the second stage, two reviewers (IR and RR) read the full texts of the remaining studies and removed any that clearly met the exclusion criteria. Any disagreements that arose were resolved through discussion.

If bibliographies of selected full texts comprised any articles consistent with the inclusion criteria, the full texts of these articles were also considered for inclusion. The number of studies at each stage of the search were recorded using the PRISMA-P flow diagram.

### Data Extraction

Data was extracted by one reviewer. The information that was extracted from articles is detailed in Textbox 3. Information on methods of PROMs development, target age range, and purposes for which PROMs were developed will be extracted by searching bibliographies for original studies describing PROM development.

**Data extracted from included articles.**

- Study design (cross-sectional, longitudinal, validation, development, interventional, review)
- Study population (number of participants)
- Type of study (quantitative or qualitative)
- Age group of participants (adult, pediatric, all ages)
- Mean age of participants, where provided
- Recruitment of patients (inpatient, outpatient, database, registry, etc)
- Setting in which patient-reported outcome measure (PROM) is administered (inpatient, outpatient)
- PROM(s) used
- Type of PROM(s) (generic, specific)
- Why PROM(s) is used (validation, outcome of intervention, prevalence, etc)
- Time points when PROM is administered (number, frequency)
- Method of administration (interview, paper, online)
- Psychometric properties of PROM assessed during the study or quoted from previous study (construct validity, content validity, internal consistency, reliability, responsiveness)
- Acceptability of PROMs to patients with cystic fibrosis as described by study authors (face validity or description of how PROMs are perceived by participants)

### Study Quality and Assessment of Risk of Bias

The COnsensus-based Standards for the selection of health Measurement INstruments (COSMIN) risk of bias checklist will be used to evaluate the methodological quality of included studies [20]. This tool has been chosen, as it specifically assesses studies that use PROMs. The tool assesses 10 measurement properties of PROMs (PROM development, content validity, structural validity, internal consistency, measurement invariance, reliability, measurement error, criterion validity, construct validity, and responsiveness). Each property is evaluated against a number of items [20].

Two reviewers will independently appraise studies using the tool. As not all studies describe all properties, only relevant areas of the COSMIN checklist will be applied to each study [20]. Reviewers will rate each item on a 4-point scale denoted as very good, adequate, doubtful, or inadequate. Results will be summarized in a table presenting the lowest score for each property [20]. Any disagreements between reviewers will be resolved through discussion.

### Analysis

A descriptive synthesis of the results will be undertaken in line with the outcomes of this study. Summary tables of characteristics of the included studies and PROMs will be presented. A description of included instruments will be given, along with the contexts in which they were used, how they were administered, and their acceptability to patients. This information will then be used to compare instruments. PROM(s) that may be suitable for inclusion into the ACFDR will be identified by considering the applicability and acceptability of instruments to a population of Australian adult and pediatric patients and caregivers. Quantitative synthesis will not be performed, as the included studies assess different outcomes.

### Ethics and Dissemination

Ethical approval is not required, as primary data was not collected. This project does not require patient or public involvement. Review results will be published in a thesis and peer-reviewed journal and will be presented at conferences. The study has been registered with the International Prospective Register of Systematic Reviews (PROSPERO); registration number CRD42019126931).

## Results

**Figure 1:**
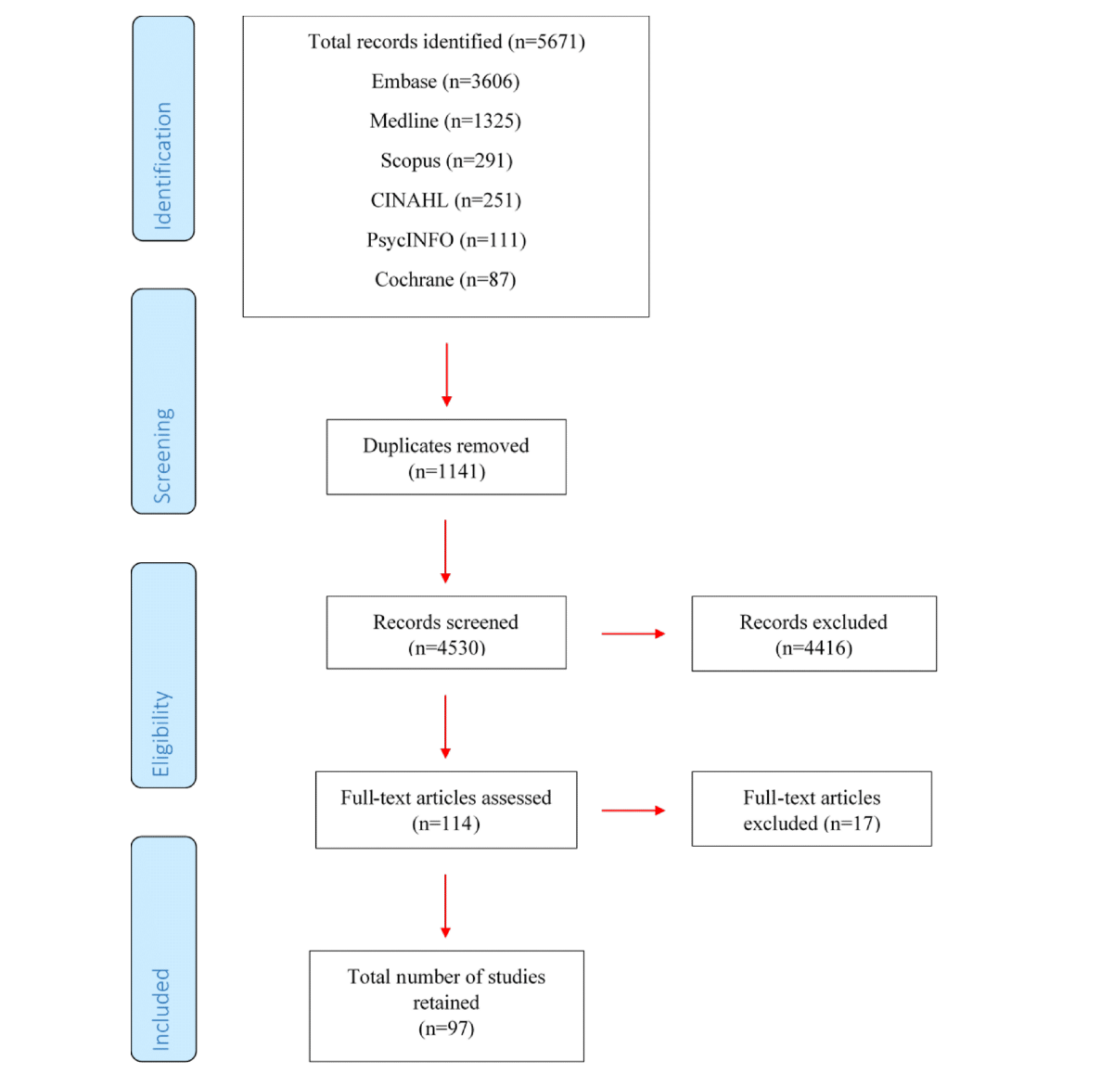
Flowchart of study selection and identification according to PRISMA-P (Preferred Reporting Items for Systematic Review and Meta-Analyses Protocol).

Reviewers conducted the search in February 2019. The final search strategy in each database is presented in Multimedia Appendix 1. The search originally yielded 5671 studies and after deleting duplicates, 4530 articles remained. The initial screen of titles and abstracts identified 114 articles that fit the inclusion criteria. Two reviewers (IR and RR) conducted a further screen of full texts and eliminated 17 articles that met the exclusion criteria. A review of bibliographies identified no further studies. The number of studies at each stage is summarized in Figure 1. Reviewers then extracted data from the remaining 97 studies. As of February 2020, reviewers have commenced data analysis.

## Discussion

To our knowledge, there is no recent systematic review describing the use of PROMs evaluating HRQOL in patients with CF. As interest in PROMs for CF grows, there is a need for a summary of all available information to understand which PROM(s) would be best suited for particular settings in CF. The proposed review aims to collate recent PROMs data used to evaluate HRQOL in patients with CF. It will identify how and in what patient populations they are administered, their effectiveness at assessing HRQOL, and their acceptability for the patient population. It will enable the identification of PROMs suitable for use in the modern Australian population with CF and in a national clinical registry setting.

This systematic review excluded randomized controlled trials (RCTs), which may limit the results regarding the extent of PROM use in CF research. However, a priori searches demonstrated that only one PROM was used in RCTs and that RCTs did not commonly provide information on the secondary outcomes of this review (administration methods, psychometric properties, or patient perspectives). Excluding RCTs may also enable a focus on observational studies, which have data collection methods more closely resembling clinical registries. Another limitation is that a search of the gray literature was not conducted, which may limit the scope of the systematic review. As preliminary gray literature searches identified no relevant sources, a formal search was not conducted.

In summary, this review will aim to identify PROM(s) that could be used to measure HRQOL in the ACFDR, a national registry collecting data from adult and pediatric patients with CF. Following identification of a suitable PROM, we plan to collect qualitative data on patient, caregiver, and clinician perceptions of the selected instrument.

## Appendix

Multimedia Appendix 1 Search strategy.

## Abbreviations

ACFDR: Australian Cystic Fibrosis Data Registry
CF: Cystic Fibrosis
CFTR: Cystic fibrosis transmembrane conductance regulator
HRQOL: Health-related quality of life
PROM: Patient reported outcome measure
