# Supplementary material for: Patient-Reported Outcome Measures in Cystic Fibrosis: Protocol for a Systematic Review
Source: JMIR Res Protoc. 2020 May 6;9(5):e15467. doi: 10.2196/15467 (PMC7240448; doi:10.2196/15467)
Supplement: Multimedia Appendix 1 [file resprot_v9i5e15467_app1.pdf]

Multimedia Appendix 1: Search strategy

Database	OVID MEDLINE
**Strategy**	**#1 OR #2 AND #3**<br>Limit English language and humans and last 10 years
#1	Patient Reported Outcome Measures/exp OR “Surveys and Questionnaires/exp OR Self Report/exp or Perception/exp OR scale.mp
#2	“Quality of Life”/exp OR QOL.mp OR “health related quality of life”. mp
#3	Cystic Fibrosis/exp
**Database**	**PsycINFO**
**Strategy**	**#1 OR #2 AND #3**<br>Limit English language and humans and last 10 years
#1	Patient reported outcome.mp OR Self Report/exp OR Client Attitudes/exp OR Questionnaires/exp OR Perception/exp OR scale.mp
#2	“Quality of Life”/exp OR QOL.mp
#3	Cystic Fibrosis/ exp
**Database**	**Scopus**
**Strategy**	**#1 OR #2 AND #3**<br>Limit English language and Publication Year 2009 – 2019 and Final Publication
#1	patient AND reported AND outcome* OR self-report* OR questionnaire OR scale OR perception
#2	quality AND of AND life
#3	cystic AND fibrosis
**Database**	**Embase**
**Strategy**	**#1 OR #2 AND #3**<br>Limit English language and humans and last 10 years
#1	Patient-reported outcome/exp OR questionnaire/exp OR self report/exp or perception/exp OR scale.mp
#2	Quality of life/exp OR QOL.mp
#3	Cystic Fibrosis/ exp
**Database**	**Cochrane**
**Strategy**	**#1 OR #2 AND #3**<br>Limit English language and humans and last 10 years
#1	Patient Reported Outcome Measures/exp OR Self Report/exp OR Survey and Questionnaries/exp
#2	Quality of Life/exp

#3	Cystic Fibrosis/ exp
**Database**	**CINAHL**
**Strategy**	**#1 OR #2 AND #3**<br>Limit English language and Publication Year 2009 - 2019
#1	“Patient-reported Outcome Measures” OR “Self Report+” OR “Patient Attitudes” OR “Questionnaires”
#2	“Quality of Life+”
#3	“Cystic Fibrosis”
